# Selected research articles from the 2017 International Workshop on Computational Network Biology: Modeling, Analysis, and Control (CNB-MAC)

**DOI:** 10.1186/s12859-018-2058-9

**Published:** 2018-03-21

**Authors:** Byung-Jun Yoon, Xiaoning Qian, Tamer Kahveci, Ranadip Pal

**Affiliations:** 1 Department of Electrical and Computer Engineering, Texas A&M University, College Station, TX 77843 USA; 2 TEES-AgriLife Center for Bioinformatics and Genomic Systems Engineering (CBGSE), College Station, TX 77845 USA; 3 Department of Computer and Information Science and Engineering, University of Florida, Gainesville, FL 32611-6125 USA; 4 Department of Electrical and Computer Engineering, Texas Tech University, Lubbock, TX 79409-3102 USA

## Introduction

The Fourth International Workshop on Computational Network Biology: Modeling, Analysis, and Control (CNB-MAC 2017) was held in Boston, Massachusetts on August 20, 2017. The workshop was organized in conjunction with the ACM Conference on Bioinformatics, Computational Biology, and Health Informatics (ACM-BCB), the flagship conference of the ACM SIGBio, as in previous years. The CNB-MAC workshop aims to provide an international scientific forum for presenting recent advances in computational network biology that involve modeling, analysis, and control of biological systems and system-oriented analysis of large-scale OMICS data.

CNB-MAC 2017 was co-chaired by Drs. Byung-Jun Yoon, Xiaoning Qian, and Tamer Kahveci. The workshop featured 14 oral presentations, which were carefully selected by the workshop chairs based on thorough reviews by the technical committee members. The final presentations at the workshop included 12 original research, one review, and one extended abstract.

With the generous support provided by the National Science Foundation (NSF), Student Travel Grants have been awarded to student authors of outstanding research papers and posters that have been invited for presentation at CNB-MAC 2017. Dr. Ranadip Pal served as the award chair for CNB-MAC 2017, and 9 awardees were selected by the award committee after a careful review of the applications and the submitted work.

## Research papers presented at CNB-MAC 2017

After the workshop, 12 papers [1–12] have been accepted for publication in the CNB-MAC 2017 partner journals after an additional round of review and revision. The following journals have partnered with CNB-MAC 2017: BMC Bioinformatics, BMC Genomics, BMC Systems Biology, and IET Systems Biology.

In [1], Foroughi pour and Dalton propose heuristic algorithms for Bayesian feature selection. Identifying biomarkers from gene expression data that may be used to discriminate between groups have been the subject of many bioinformatics studies. One may be interested in finding effective combinations of features – or “marker families” – rather than individual features, when prominent individual markers are not present or if the primary goal is to detect potential interactions between markers. Considering all possible combinations of markers would be computationally intractable, and the authors demonstrate that the proposed feature selection algorithms can address this issue, and develop a method which outperforms existing algorithms.

Constructing predictive models that can accurately predict the drug sensitivity for individual cancer cell lines based on genomic features can have significant impacts on precision medicine. Matlock et al. [2] investigates the problem of stacking predictive models that may incorporate various types of data to enhance the prediction performance. By comparing individual and stacked models, Matlock et al. report that stacking models trained on heterogeneous datasets have important advantages over stacking different models trained on the same dataset, enhancing the overall prediction accuracy and reducing the bias inherent in predictive models such as Random Forest.

The analysis of variance heterogeneity in genome wide association studies (vGWAS) has emerged as a new approach for investigating the genetic origins of various traits, especially those that may be associated with various diseases. It has been shown that vGWAS may complement conventional GWAS, by enabling the detection of genetic loci where significant change in variance heterogeneity may be introduced as a result of potential gene-gene or gene-environment interactions. In [3], Al Kawam et al. present a novel simulation procedure that could be used for the quantitative performance assessment of vGWAS analysis methods. The utility of the proposed framework and algorithm is demonstrated based on several scenarios, where the evaluation results are used to highlight the limitations of current analysis techniques and the challenges that need to be addressed in the future.

Katiyar et al. [4] investigate the problem of computationally determining the composition of heterogeneous cancer tissues. Heterogeneity in cancer tissues is known to critically affect the survival, growth, and metastasis of cancer cells, hence accurate estimation of the composition of a heterogeneous cancer tissue may ultimately lead to more effective cancer therapeutics. In [4], Katiyar et al. propose a Bayesian approach to tackle the composition prediction problem. The proposed algorithm takes advantage of high quality data obtained by single cell line cell-by-cell observation methods for training the model, which can be used for estimating the composition of heterogeneous cancer cell mixtures from low cost measurement data. The algorithm is analyzed and validated based on synthetic as well as experimental data.

In [5], Ni et al. provide a comprehensive review of reciprocal graphs (RGs) and recent developments in RG-based approaches for modeling biological networks. A reciprocal graph is a graph that can consist of both directed and undirected edges, with the restriction that nodes in the same “path component” (defined as the set of nodes that are all connected by an undirected path) cannot be connected by directed edges. As RG models can model regulatory relationships in ways that allow cycles, they are suitable for modeling molecular networks with feedback mechanisms and have the potential to yield models that are biologically more interpretable. Ni et al. show how the RG approach can be extended to model networks by integrating diverse molecular data and demonstrate how its application to TCGA (The Cancer Genome Atlas) ovarian cancer data leads to interesting findings.

Haplotype assembly aims to reconstruct the haplotypes for a chromosome from a collection of sequence fragments obtained from high-throughput sequencing. In [6], Hashemi et al. propose a novel haplotype assembly algorithm, called AltHap, by formulating the haplotype assembly problem as a sparse tensor decomposition problem. Based on the tensor decomposition framework, AltHap iteratively assembles the haplotypes by exploiting the structural properties of the sparse tensor. The proposed algorithm is fairly general and can be applied to haplotype assembly of diploids as well as biallelic and polyallelic polyploids. Evaluation results show that AltHap favorably compares to other existing methods for haplotype assembly of diploids, while significantly outperforming them for haplotype assembly of polyploids.

Identifying genetic markers with both the marginal and epistatic effects has been one of the critical challenges for better understanding of living systems and more accurate phenotypic prediction. Many heuristic measures, such as correlation and mutual information, have been adopted to estimate statistical association among pairs of features and the outcome for this purpose. The existing literature only provides empirical performance evaluation but without solid theoretical guarantees or clear understanding on which essential information or interaction among features is captured by these methods. In [7], Xu et al. establish rigorous mathematical theories for feature screening and selection approaches with the consideration of interactive effects under logistic regression models for genotype-phenotype association. The authors prove that the proposed information theoretic synergistic effect measure can approximate the quadratic functions of the coefficients of the interaction terms in logistic regression and it can be estimated with a tight upper bound of the estimation error, demonstrated by both simulated and real-world GWAS datasets.

Phenotype classification based on gene expression data often suffers from limited specificity, as the expression measurements are typically averaged across cells and the pathway dynamics remain hidden. Should single-cell expression trajectories be available such that the measurements are made at a sufficiently high rate to capture the regulatory timing in gene regulatory networks, the classification accuracy may be significantly improved. Karbalayghareh et al. [8] investigates the performance of intrinsically Bayesian robust classifiers for discriminating between wild-type and mutated gene regulatory networks based on single-cell gene expression trajectories, where it is assumed that the network model is only partially known. The study reveals how the length of the trajectories, the amount of uncertainty in the underlying model, as well as other parameters affect the classification error.

In [9], Hall-Swan et al. compares popular network clustering methods for decomposing a protein-protein interaction (PPI) network into non-overlapping network modules. Clustering PPI networks provides an efficient means of analyzing the organization of PPI networks and may be used to detect novel functional modules that are embedded in the network. In this work, the authors examine how preprocessing the PPI network by removing and reweighting the edges based on the diffusion state distance (DSD) – referred to as “detangling” the network – affects the network clustering performance. It is demonstrated that, in most cases, clustering PPI networks after detangling them based on DSD yields clusters that are biologically more meaningful.

Miannay et al. [10] tackles the problem of integrating gene expression profiles (GEPs) and large-scale biological networks by considering the underlying network logic. In the proposed approach, the logic underlying the biological network of interest is first represented using answer set programming (ASP). Subcomponents of the logic network are then extracted by solving the optimal graph coloring problem, where coloring constraints are set based on the regulatory logic. These components are compared with the GEP and those components whose configurations have maximal similarity with the observed expression profiles are selected. The proposed approach was applied to the analysis of multiple myeloma gene expression data, which revealed functional subgraphs that may be associated with the disease.

Cheng et al. [11] propose a novel subcellular network module identification algorithm, called SMILE (Subcellular Module Identification with Localization Expansion). Unlike many existing network clustering methods, which predict functional modules in PPI networks based on network topology, SMILE incorporates subcellular location information to enhance module prediction. The algorithm first predicts subcellular network modules in separate cell compartments and highly overlapping modules are merged to obtain “super-modules”. They demonstrate that the predicted super-modules better correspond to known protein complexes and pathways, compared to those detected by other popular clustering methods that do not consider subcellular localization.

While most network module identification methods are unsupervised, semi-supervised methods have the potential to significantly improve the prediction results by incorporating additional constraints that may be derived from prior biological knowledge. In [12], Liu et al. introduce a novel semi-supervised functional module detection method called PCNMTF (pairwise constrained non-negative matrix tri-Factorization). PCNMTF extracts pairwise constraints between proteins based on whether they participate in known protein complexes. These constraints are subsequently used to define a regularization term in the optimal non-negative matrix tri-factorization problem, in order to accurately identify functional modules in a manner that balances the topological features in the PPI network and the prior biological knowledge at hand. Assessment on both synthetic and real biological networks shows that PCNMTF yields more accurate predictions, outperforming previous methods.
